# Prenatal serotonin reuptake inhibitor (SRI) antidepressant exposure and serotonin transporter promoter genotype *(SLC6A4)* influence executive functions at 6 years of age

**DOI:** 10.3389/fncel.2013.00180

**Published:** 2013-10-11

**Authors:** Whitney M. Weikum, Ursula Brain, Cecil M. Y. Chau, Ruth E. Grunau, W. Thomas Boyce, Adele Diamond, Tim F. Oberlander

**Affiliations:** ^1^Pediatrics, Child and Family Research Institute, University of British ColumbiaVancouver, BC, Canada; ^2^Developmental Cognitive Neuroscience Lab, Psychiatry, University of British ColumbiaVancouver, BC, Canada

**Keywords:** serotonin, executive function, childhood, prenatal exposure, SRI, *SLC6A4* genotype, depression

## Abstract

Prenatal exposure to serotonin reuptake inhibitor (SRI) antidepressants and maternal depression may affect prefrontal cognitive skills (executive functions; EFs) including self-control, working memory and cognitive flexibility. We examined long-term effects of prenatal SRI exposure on EFs to determine whether effects are moderated by maternal mood and/or genetic variations in *SLC6A4* (a gene that codes for the serotonin transporter [5-HTT] central to the regulation of synaptic serotonin levels and behavior). Children who were exposed to SRIs prenatally (SRI-exposed *N* = 26) and non-exposed (*N* = 38) were studied at age 6 years (*M* = 6.3; *SD* = 0.5) using the Hearts & Flowers task (H&F) to assess EFs. Maternal mood was measured during pregnancy (3rd trimester) and when the child was age 6 years (Hamilton Depression Scale). Parent reports of child behavior were also obtained (MacArthur Health & Behavior Questionnaire). Parents of prenatally SRI-exposed children reported fewer child externalizing and inattentive (ADHD) behaviors. Generalized estimate equation modeling showed a significant 3-way interaction between prenatal SRI exposure, *SLC6A4* variant, and maternal mood at the 6-year time-point on H&F accuracy. For prenatally SRI-exposed children, regardless of maternal mood, the H&F accuracy of children with reduced 5HTT expression (a short [S] allele) remained stable. Even with increasing maternal depressive symptoms (though all below clinical threshold), EFs of children with at least one short allele were comparable to children with the same genotype whose mothers reported few if any depressive symptoms—in this sense they showed resilience. Children with two long (L) alleles were more sensitive to context. When their mothers had few depressive symptoms, LL children showed extremely good EF performance—better than any other group. When their mothers reported more depressive symptoms, LL children's EF performance was worse than that of any other group. In the face of a mother with a more depressed mood, EFs were best preserved in children prenatally exposed to SRIs and with at least one short *SLC6A4* allele. Yet, prenatally-exposed LL children hold out promise of possibly superior EF if their mother's mood remains euthymic or improves.

## Introduction

Serotonin (5-HT) and its multiple receptors are highly expressed in prefrontal cortex (PFC) and play key roles in influencing complex cognition and resilience to stress (Canli et al., 2005; Lesch, 2007; Reuter et al., 2007; Homberg and Lesch, 2011). Dense projections of 5-HT neurons into prefrontal regions (Preece et al., 2004), and a wide distribution of 5-HT receptors and 5-HT transporter sites in PFC (Varnäs et al., 2004) contribute to 5-HT's role in cognition (King et al., 2008). Critical cognitive capacities that rely on PFC and related structures (Miller and Cohen, 2001; Braver et al., 2002; Petrides, 2005; Champod and Petrides, 2007; Zanto et al., 2011) are termed executive functions (EFs), and include abilities to (1) focus, sustain and shift attention (executive attention), (2) resist the pulls and temptations of external stimuli, our emotions, or engrained behavioral tendencies, inhibit acting impulsively, taking a moment to make a more considered response (inhibitory control), (3) hold information in mind and work with it, such as updating one's thinking or planning when given new information, considering alternatives, or mentally relating pieces of information to one other (working memory), and (4) creative problem-solving, flexibly adjusting to changed demands, priorities, new obstacles or opportunities (cognitive flexibility; Miyake et al., 2000; Diamond, 2013). Not surprisingly, good EFs are critical for all aspects of life, including mental and physical health and success in school and in life (Moffitt et al., 2011; Diamond, 2013). For example, childhood EFs predict school readiness and success in math and reading throughout all school years from kindergarten through university better than does IQ, even when controlling for SES (Bull and Scerif, 2001; Blair, 2002; Riggs et al., 2004; Blair et al., 2005; Gathercole et al., 2005; Blair and Razza, 2007).

5-HT plays a critical role in brain development (Kalueff et al., 2010; Olivier et al., 2011). In animal models, developmental shifts in central 5-HT signaling shape early cognitive capacities setting pathways for learning and behavior later in life (see for review Kalueff et al., 2010). Little, however, is known about how developmental changes in 5-HT influence early cognitive development in humans during childhood.

The increasing use of serotonin reuptake inhibitor (SRI) antidepressants to manage maternal mood disorders during pregnancy (Cooper et al., 2007) raises critical questions about the impact of prenatal altered central 5-HT levels on the development of systems that regulate attention, working memory, and self-control (i.e., EFs) in childhood (Kalueff et al., 2010; Hanley and Oberlander, 2012). SRIs primarily act by blocking reuptake of serotonin transporter protein (5-HTT), thereby increasing how much, and how long, extracellular 5-HT remains active and available. SRIs readily cross the placenta and the blood-brain barrier (Kim et al., 2006) altering fetal central 5-HT levels (Laine et al., 2003). Prenatal SRI exposure affects (1) fetal (Salisbury et al., 2009; Mulder et al., 2011) and newborn neurobehavior (Moses-Kolko et al., 2005), (2) neonatal stress regulation (Oberlander et al., 2002, 2005), (3) shifts language perception during the first year of life (Weikum et al., 2012), and (4) is associated with emotional regulation in toddlers (Oberlander et al., 2010).

Why some, but not all, children are affected by prenatal SRI exposure is still a central and pressing question (Hanley and Oberlander, 2012). In the early school years prenatally exposed children appear to have typical language development, behavior and IQ (Nulman et al., 2002). However, not all outcomes can be specifically attributed to prenatal antidepressant exposure. Distinguishing the concurrent impact of pre and postnatal maternal mood disturbances remains challenging (Oberlander et al., 2010).

The pre-synaptic membrane-bound serotonin transporter protein (5-HTT)—the very target of SRI antidepressants—is central to the regulation of intra-synaptic 5-HT. Allelic variations in *5-HTTLPR* (*SLC6A4*) influences gene transcription and the amount of 5-HT available at postsynaptic sites (Lesch et al., 1996). The short (S) variant is associated with reduced gene transcription and reduced levels of 5-HTT protein, with an ~50% reduction in 5-HT reuptake compared to the long (L) variant (Heils et al., 1996; Homberg et al., 2007a). Reduced 5-HTT protein availability and 5-HT reuptake results in a higher effective “serotonin dose.”

Homozygosity for the short (S) allele is associated with increased stress sensitivity and risk for emotional disturbances including anxiety and depression but better EFs (not unlike what has been found for the COMT- MET genotype (Goldman et al., 2005; Diamond, 2011). In combination with early life stressors, the short allele has been widely studied as an important risk factor for mental illness later in life (Caspi et al., 2003; Kendler et al., 2005; Lesch, 2007). For example, adolescents who encountered adversity in childhood and are homozygous for the short allele of *5-HTTLPR* have a heightened sensitivity to potential negativity and threat in the environment and are more prone to anxiety and depression (Owens et al., 2012). In animal models, increased 5-HT levels secondary to 5-HTT blockade at developmentally sensitive time periods (akin to a human 3rd trimester) causes permanent axonal connection deficits in the somatosensory cortex (Homberg et al., 2010), the lateral geniculate nucleus (Gaspar et al., 2003), and altered neuronal dendritic branching, elongation and pruning (Homberg et al., 2010; Liao and Lee, 2011; Olivier et al., 2011; Simpson et al., 2011; Zheng et al., 2011). Beyond the newborn period, SRI-exposed animals demonstrate *decreased* 5-HT levels—possibly via prolonged activation of inhibitory receptors (i.e., 5-HT_1a_; Hensler, 2006). This might underlie the reduced novelty investigation, poorer motor performance (Lee and Lee, 2012), increased anxiety in conflict tasks and anhedonia (Ansorge et al., 2004, 2008; Popa et al., 2008) reported in fluoxetine-exposed mice. Adults with two short *5-HTTLPR* alleles consistently outperform those with one or two long alleles on measures of EFs such as the Wisconsin Card Sorting test (Borg et al., 2009) and go/no-go tests (Roiser et al., 2007), they also show brain patterns consistent with better EFs (Enge et al., 2011). Conversely, the L-allele of the 5-*HTTLPR* gene is associated with poor EFs including impulsivity, inattention, and working memory deficits (see the meta-analysis by Gizer et al., 2009). Together these findings support the notion that changes in transcriptional activity associated with allelic variations in the 5-*HTTLPR* gene and presumably reflecting alterations in central serotonin levels, influence EFs in the mature adult brain.

Beyond genetic variations, experimental manipulations of central serotonin levels in adults also appears to affect cognitive functions. Acute SRI administration to healthy adults has been shown to improve verbal fluency, a measure of EFs requiring memory of words, inhibitory control to avoid repeating words, and cognitive flexibility to switch to different paths and strategies for coming up with words (Schmitt et al., 2001). Although reduced 5-HT, using an acute tryptophan depletion (ATD) model with healthy volunteers, has been found to improve focused attention (Schmitt et al., 2000; Evers et al., 2006), enhanced EF performance and reduced impulsivity have also been found in some animal models of SRI exposure (e.g., Sasaki-Adams and Kelley, 2001) but not all (e.g., Valluzzi and Chan, 2007). Importantly, *5-HTTLPR* genotype and SRI exposure do not affect, or inconsistently affect, non-EF cognitive abilities such as recall and recognition memory and mental rotation (e.g., 5-*HTTLPR* genotype: Roiser et al., 2006; Mannie et al., 2009, SRI exposure: Harmer et al., 2002; Siepmann et al., 2003; Riedel et al., 2005).

An acute pharmacological exposure to an SRI or dietary depletion of tryptophan in a mature brain may not result in the same consequences as chronic prenatal SRI exposure and the associated long-term changes in prenatal 5-HT signaling that occurs with such exposure across developmentally sensitive periods of brain growth (Ansorge et al., 2004). To date, studies focusing on the effects of prenatal SRI exposure have typically sought to examine the consequences of what is generally considered increased developmental serotonergic tone. However, in humans the developmental course or behavioral consequences that might follow prenatal SRI exposure (i.e., downstream lower serotonergic tone) is not known. Given the developmental role of 5-HT, it is conceivable that prenatal changes in 5-HT either via genetic variations or prenatal SRI exposure might influence early cognitive development in humans.

To further understand the developmental impact of prenatal SRI exposure on early cognitive development, we studied whether prenatal exposure to SRI antidepressants or maternal mood affects core cognitive skills (EFs) in early childhood, controlling for prenatal maternal mood. Secondarily, we also sought to examine whether changes in EFs are moderated by the child's *SLC6A4* genotype, reflecting genetic variations in the capacity to control serotonergic tone that may influence the impact of exposure to maternal mood or SRIs. Given fetal changes in 5-HT signaling secondary to prenatal SRI exposure, and the literature showing improved cognitive function among S carriers, we expected that antidepressant exposure and reduced *SLC6A4* transcription (at least one short [S] allele) at a developmentally sensitive time (i.e., *in utero*) would be associated with improved EF capacity in early childhood, while elevated maternal depressive symptoms would have an opposing effect at 6 years of age.

## Materials and methods

### Participants

Children in this study are part of a longitudinal cohort study examining the effects of prenatal exposure to SRIs and maternal mood disturbances in 98 mothers recruited during their second trimester of pregnancy. Approval was obtained from the University of British Columbia Ethics Board and the Children's and Women's Health Centre of British Columbia Research Review Committee. Written informed parental consent was obtained to follow the development of these children. All mothers, regardless of their mood or medication status, were physician-referred or self-referred from the Reproductive Mental Health Clinic at British Columbia Women's Hospital and Health Centre (a tertiary-care service), community midwife clinics or family physician practices in the greater Vancouver metropolitan area. All SRI-treated mothers had started taking medications based on clinical need, had a diagnosis of a mood disorder, and were already taking antidepressant medications at the time of conception. Women in the non-SRI group had a range of mood symptoms at the time of recruitment as assessed by the Hamilton Rating Scale for Depression (HAM-D; see Table 1). Of the original 98 mothers, 4 withdrew before the baby was born and another 4 withdrew before the end of the child's first year. At 6 years, an additional 26 children were unavailable for study (22 families had moved and 4 mothers had withdrawn by 3 years). At the time of this study, 64 children (26 prenatally SRI-exposed and 38 non-exposed) were seen at mean age 6.3 years (*SD* = 0.51 years). From this sample, 25 exposed and 32 non-exposed had both prenatal maternal mood scores and samples of the child's blood available for genotyping.

**Table 1:** **Maternal characteristics**.

	**Non-exposed (***n*** = **38**)**	**SRI-exposed (***n*** = **26**)**	***T***	***p*-value**
Prenatal HamA (mean) (*SD*) (*n* = 35 non-exposed and 25 SRI-exposed)	5.5 (4.75)	9.58 (6.75)	−2.8	0.007
Prenatal HamD (mean) (*SD*) (*n* = 34 non-exposed and 25 SRI-exposed)	3.64 (4.26)	8.54 (6.28)	−3.66	0.001
Maternal smoking during pregnancy	0	0		0
Maternal alcohol consumption (drinks in pregnancy) (*n* = 37 non-exposed)	2.49 (4.49)	3.69 (8.35)	−0.74	0.462
Maternal age at birth (years)	33.21 (4.93)	31.42 (4.6)	1.45	0.151
Maternal education (years)	17.8 (2.71)	15.27 (2.39)	3.83	0.001
**MATERNAL *SLC6A4* GENOTYPE (n's)**				
LL	10	7		0.704
1S	27	18		

### Child mental health symptomatology

Measures of child mood and behavior were obtained from the mental health symptomatology section of the MacArthur Health and Behavior Questionnaire (HBQ; Boyce et al., 2002; Essex et al., 2002) that was completed by maternal report (HBQ-P) and yielded measures of internalizing, externalizing and Attention-Deficit and Hyperactivity Disorder (ADHD) behaviors for each child. The HBQ was derived from the Ontario Child Health Study measure designed to map onto DSM-III-R symptom criteria (Boyle et al., 1993). The HBQ-P has strong psychometric properties and has been used to assess child mental health across multiple ages from 4.5 years into adolescence (Ablow et al., 1999; Essex et al., 2006; Shirtcliff and Essex, 2008) The mental health scales have been shown to discriminate groups of children with and without signs of early psychopathology (Luby et al., 2002).

The HBQ-P, administered in questionnaire format, assesses symptoms ranging from “never or not true” to “often or very true.” Symptoms in three domains were analyzed: (1) ADHD symptoms consist of items indexing inattention, impulsivity, and hyperactivity. (2) Externalizing symptoms consist of items indexing oppositional defiant behaviors and conduct problems. (3) Internalizing symptoms consist of items indexing symptoms of depression, separation anxiety, and generalized anxiety. In addition to mean symptom level, the percentage of children above clinical cutoffs was examined. Clinical cutoffs for parent reported ADHD, externalizing, and internalizing symptoms (1.2, 0.68, 0.71, respectively) were set based on previous analysis of the HBQ-P (Lemery-Chalfant et al., 2007) with children of approximately the same age as in the present study.

### EF tasks

EFs were assessed using the H&F task, a computerized measure that has been validated with children 4–13 years of age and with adults (Davidson et al., 2006; Diamond et al., 2007). This task assesses inhibition, working memory and cognitive flexibility. A stimulus appears to the right or left of a computer screen on every trial. On Block 1 of the task (the congruent block), participants have only to do what comes naturally (i.e., pressing on the same side as the stimulus); no EFs are taxed. On Block 2 (the incongruent block), participants had to resist that prepotent response and instead press on the side opposite the stimulus. On Block 3 (the mixed block), the two types of trials are randomly intermixed, requiring remembering both rules and mentally translating “same [or opposite] side” into “right [or left] hand,” and flexibly switching between the two rules, inhibiting one to apply the other.

The children came to the study center mid-morning and first performed a warm up task (about 5 min). During the task, children were told to respond to a stimulus as fast as they were able and this gave them practice with the computerized set-up. The children then performed the H&F task (about 10 min). Practice trials were given before both the congruent and incongruent blocks (see Davidson et al., 2006; Diamond et al., 2007). In both blocks, children were given up to 6 s to respond, and 10 s in the mixed block. Responses > 2000 ms were considered incorrect (inattentive) and those < 250 ms, impulsive. Both responses were excluded. Five trials out of 1860 trials were > 2000 ms (0.26%), and there were no trials < 250 ms (out of 1860 trials in total). Outlier trials were removed by using a lower and upper threshold of 2 standard deviations from the mean RT per trial type per block and per subject.

Two dependent measures were tabulated for each block (i.e., Congruent, Incongruent, etc): (1) correct responses or *accuracy* (% correct = #correct/[# trials]) and (2) Reaction time or *speed of response* (reaction time, RT > 250 msec for correct trials only). Reaction time was a *Choice RT* tabulated every time a stimulus appeared during the three-block task and the stimulus appeared at random intervals (one button used) (Kail and Salthouse, 1994).

### Maternal mood

Maternal mood was assessed during the third trimester of pregnancy (mean 33.8 week; *SD* 1.25 weeks), and again at the 6-year timepoint using the HAM-D; (Hamilton, 1960), a 21-item clinician-rated measure of depressive symptoms with a score ranging from 0 to 63.

### *SLC6A4* genotyping

Genomic DNA was extracted from neonatal whole blood samples using the Flexigene DNA Blood Kit (Qiagen, Valencia, California). The S and L alleles of *SLC6A4* were identified as previously described in (Lesch et al., 1993). Polymerase chain reaction was performed with oligonucleotide primers flanking the polymorphism (corresponding to nucleotide positions -1416 to -1397 [stpr5, 5_- GGCGTTGCCGCTCTGAATGC] and -910 to -888 [stpr3, 5_-GAGGGACTGAGCTGGACAACCAC]) of the 5_-flanking regulatory region of *SLC6A4* to generate a 484-bp (S short allele) or a 528-bp (L long allele) polymerase chain reaction product. Polymerase chain reaction amplification was performed in a final volume of 30 μ L with 50 ng of genomic DNA, 2.5mM deoxyribonucleotides (dGTP/7-deaza-2_-dGTP = l/l), 0.1 μ g of sense and antisense primers, 10 mM Tris hydrochloride (pH 8.3), 50 mM potassium chloride, 1.5 mM magnesium chloride, and 1 U of Taq DNA polymerase. For quality control, 5% of the samples were randomly chosen to be retested and their genotypes were consistent with previous results.

### Statistical analyses

Two separate analytic approaches were used to study behavioral outcomes. To analyze child behavioral differences using maternal report, a multivariate analysis of covariance (MANCOVA) was used to examine group (SRI exposed vs. non exposed) differences in child behavior, with child age (at the time of the 6 year study, prenatal (3rd trimester) and postnatal (6 year) maternal mood as covariates. Maternal mood was used as a continuous measure to allow us to account for the wide range of depressive symptoms observed among both SRI-exposed and non-exposed groups. Across time, some mothers in our untreated group became depressed and some crossed over to the SRI treated group. Maternal mood measures at both time points (prenatally and at the 6 year study) helped to account for these changes.

General Estimating Equation (GEE) modeling was used to examine group (SRI exposed vs. non-exposed) and genotype (LL vs. at least one S allele) in relation to each of the three computerized H&F conditions. Due to a limited number of children with SRI exposure and two short alleles (*n* = 7), children with at least one short allele (LS and SS) were grouped together to yield the ≥1 S allele group. With the GEE approach we were able to examine main effects (SRI exposure and genotype) and interactions simultaneously. The role of genotype was examined as a possible moderator of the effects of prenatal exposure on EFs by comparing performance for each EF task block and interactions between exposure group and *SLC6A4* genotype (LL vs. at least one S allele [SS or LS]), accounting for pre- and post-natal maternal mood. GEE extends the generalized linear modeling to allow for analysis of repeated measurement of accuracy (a binomial dependent variable). GEE analyses were performed using SPSS Statistics 18. All *p*-values less than 0.05 were considered significant.

## Results

Demographic and behavioral outcomes for the mothers and their children are presented in Tables 1, 2. With the exception of maternal education, maternal mood prenatally and at the 6 year study, child age at the time of the study (*p* = 0.03), and the children's 5-min APGAR scores (*p* = 0.026), no significant group differences (SRI exposed vs. non-exposed) were observed. As SRI-exposed children were older than the non-exposed children at the time of the study, child age was included as a covariate in the analyses. While the 5-min APGAR scores were statistically different between groups, the clinical impact of these differences (i.e., between scores of 9.13 vs. 8.73, Table 1) would not reflect a significant difference in outcome and thus were not included in further analyses. We did not include maternal education as a covariate as all mothers had high levels of education.

**Table 2:** **Child characteristics**.

**Child characteristics**	**Non-exposed (***n*** = **38**)**	**SRI-exposed (***n*** = **26**)**	***T***	***p*-value**
Gestation age at birth (mean weeks) (*SD*)	40.0 (1.35)	39.4 (1.63)	1.54	0.128
Birth weight (grams) (*SD*)	3531 (470)	3317 (480)	1.77	0.081
Birth length (cm) (*SD*)	51.7 (2.8)	50.71 (2.54)	1.48	0.143
Head circumference (cm) (*SD*)	35.0 (1.36)	34.42 (1.27)	1.69	0.095
Apgars score (1 min)	8.03 (1.6)	7.65 (1.55)	0.93	0.358
Apgars score (5 min)	9.13 (0.53)	8.73 (0.87)	2.28	0.026
Sex (m:f)	18:20	10:16	−0.7	0.488
*SLC6A4* genotype (n's)				0.946
LL	11	9		
1S	23	16		
Age at study (yr) (*SD*)	6.22 (0.55)	6.51 (0.45)	−2.28	0.03
**Child mental health symptomatology**			***F***	***p*-value**
Internalizing symptoms^^^ *Composite of depression, separation anxiety, and generalized anxiety scores*	0.33 (0.25)	0.33 (0.22)	0.253	0.617
% above clinical threshold	6.3	8		
Externalizing symptoms^^^ *Composite of oppositional defiant behaviors and conduct problem scores*	0.29 (0.22)	0.23 (0.17)	5.831	0.019
% above clinical threshold	6.3	0		
ADHD Symptoms^^^ *Composite of inattention, impulsivity, and hyperactivity scores*	0.67 (0.39)	0.47(0.37)	4.954	0.03
% above clinical threshold	9.4	0		

^ Controlling for maternal mood (prenatal and 6 year) and child age.

### Child mental health

In SRI-exposed children, significantly fewer ADHD (*p* = 0.03) and disruptive externalizing symptoms (*p* = 0.019) were reported by parents, after adjusting for child age, 3rd-trimester maternal mood and maternal mood at the time of the study. No differences in internalizing behaviors were found between exposure groups (Table 2). Maternal depression symptoms were associated with increased report of externalizing (*r* = 0.314; *p* = 0.01) and ADHD behaviors (*r* = 0.251; *p* = 0.039). In separate GEE models, internalizing and externalizing behaviors, respectively, were not predictive of EF performance, regardless of concurrent mother's mood.

### EF task (hearts and flowers)

To examine the effect of SRI exposure, *SLC6A4* variant and maternal mood on EF performance (accuracy and reaction time), a GEE model was run separately on each of the three blocks of the EF task. SRI exposure (yes/no) and *SLC6A4* variant (LL vs. at least one S) were factors. Trials was a repeated within-subject variable, and maternal mood measures (prenatal and at the 6 year study) and child age at test day were covariates. The outcome was either accuracy (% correct response) or RT (in milliseconds) on the EF task.

### Reaction time

**Figure 1:**
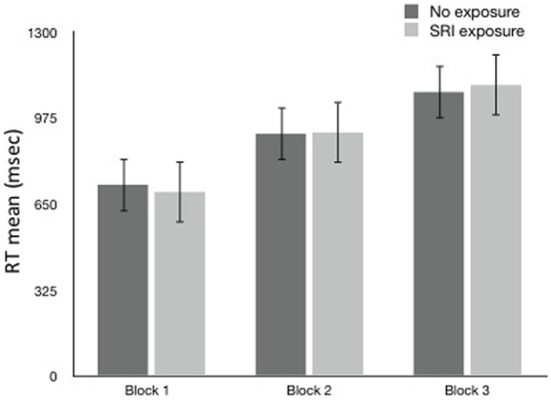
**Reaction Time (ms ± sem) by block and SRI Exposure**.

Overall, no SRI exposure group differences in RT (Figure 1) were found in any of the 3 blocks. RT increased with increasing task difficulty, but that did not differ by SRI exposure or genotype (LL vs. ≥1 S allele). In a separate analysis of Choice RT task, older children were faster (*B* = −35.5, *p* = 0.048). To control for speed of responding faster, Choice RT was added to an overall GEE RT model as a covariate. There was still no significant effect for SRI exposure or SLC6A4 genotype (LL vs. ≥1 S allele) in the GEE RT model.

### Accuracy

**Figure 2:**
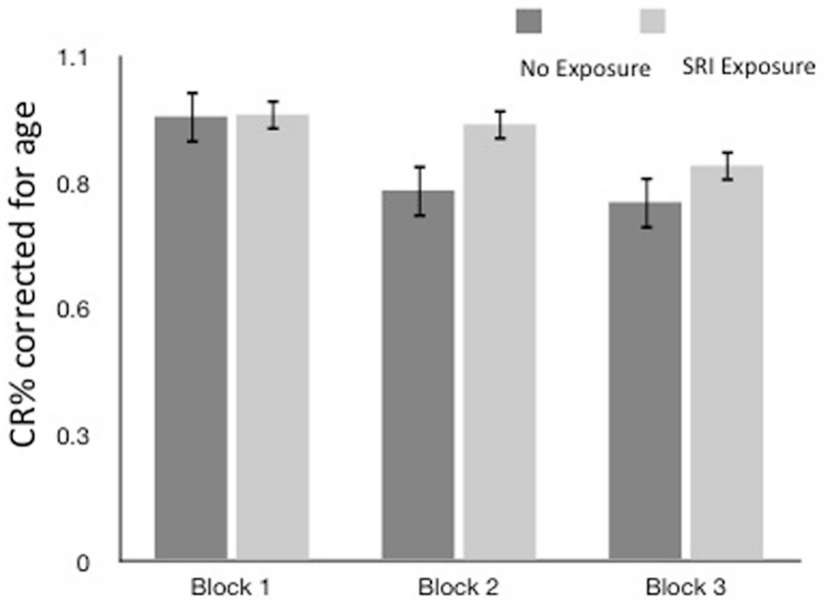
**Accuracy (CR % ± SEM) by block and SRI exposure (CR% corrected for age)**.

Overall, no SRI exposure group differences in accuracy (Figure 2) were found on block 1 or 2. Differences emerged in the most difficult third block where exposed children showed higher accuracy (suggesting better cognitive flexibility). However, the results were not significant when controlling for child age. Not surprisingly, older children performed better on the EF test (*p* = 0.004); the results of the models were adjusted for child age. Maternal depressed mood at 3rd trimester contributed, but not statistically significantly (*p* = 0.064, *OR* = 0.943).

With each block, differences in accuracy between allelic variations and exposure groups began to emerge, but only in block 3, with accuracy as the dependent variable, a significant main effect for *SLC6A4* genotype, child age and maternal mood emerged, as well as a significant 3-way interaction between prenatal SRI exposure, *SLC6A4* variant, and maternal mood at age 6 years in the GEE model (Table 3), controlling for child age.

**Table 3:** **GEE model results (reflecting slope) showing the effect of SRI exposure, *SLC6A4* variant, and maternal mood on EF task accuracy**.

**Parameter**	B^*^	**95% Wald confidence interval**		**Wald χ 2**	***p*-value**
		**Lower**	**Upper**		
SRI exposure	0.516	−0.236	1.268	1.807	0.179
*SLC6A4* LL	1.051	0.263	1.838	6.838	**0.009**
Third trimester mood (HAMD)	−0.059	−0.122	0.003	3.429	0.064
Maternal Mood Study Day (HAMD)	0.099	0.035	0.163	9.295	**0.002**
Child age at study day	0.682	0.221	1.143	8.398	**0.004**
Prenatal mood ^*^ SRI non-exposed ^*^ *SLC6A4* LL	−0.047	−0.162	0.068	0.632	0.427
Prenatal mood ^*^ SRI non-exposed ^*^ *SLC6A4* ≥ 1 s	0.056	−0.024	0.136	1.884	0.17
Prenatal Mood ^*^ SRI exposed ^*^ *SLC6A4* LL	−0.02	−0.094	0.055	0.271	0.602
Six year maternal mood ^*^ SRI non-exposed ^*^ *SLC6A4* LL	−0.171	−0.261	−0.082	14.098	**<0.001**
Six year maternal mood ^*^ SRI non-exposed ^*^ *SLC6A4* ≥ 1 s	−0.139	−0.216	−0.061	12.237	**<0.001**
Six year maternal mood ^*^ SRI exposed ^*^ *SLC6A4* LL	−0.092	−0.171	−0.014	5.29	**0.021**

B

* is the non-standardized regression coefficient. Bold value indicates *p* < *0.001*.

**Figure 3:**
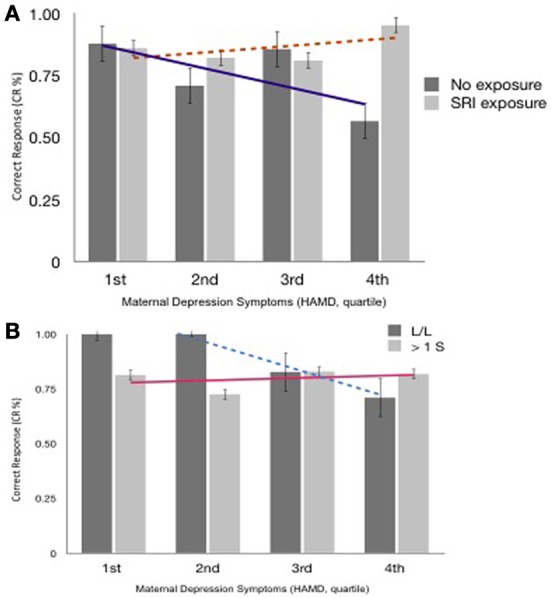
**(A)** Accuracy (% Correct Response, Block 3 ± SEM), SRI exposure & Maternal Depressed Mood at 6 years postpartum (quartile). Trend lines reflect differences in CR% across maternal mood between exposure groups. **(B)** Accuracy (CR%, Correct Response, Block 3 ± SEM), *SLC6A4* Allelic variations & Maternal Depressed Mood at 6 years postpartum (quartile). Trend lines reflect differences in CR% across maternal mood between allele groups.

When the mother's current depressed mood symptoms were relatively low (measured on test-day), EF performance did not differ with the presence of prenatal SRI exposure (Figure 2) or by child genotype (Figure 3A, using maternal mood grouped by quartiles to illustrate the GEE results). However, the more depressed the mother was currently, the more performance between the groups began to diverge. In the face of higher depressive maternal symptoms (4th quartile), EF performance of children with no prenatal SRI exposure was poor. Accuracy was significantly and inversely related to how depressed their mother was currently (*B* = 0.099; 95% CI [0.035–0.163]; χ^2^ = 9.295; *p* = 0.002) and this was particularly true for children with the LL variant (*B* = −0.092; 95% CI [−0.171–−0.014]; χ^2^ = 5.29; *p* = 0.021). Namely, children prenatally exposed to SRIs and with at least 1 S allele and high concurrent maternal depressive symptoms showed no decrement in accuracy. However, among children with LL variant of SLC6A4, accuracy was worse in children with symptomatic mothers (3rd and 4th quartiles) compared with those with less symptomatic mothers. In contrast, children with = 1 S allele had relatively stable performance regardless of mothers' depressive mood states (Figure 3B).

## Discussion

On a test of EFs (H&F; requiring inhibition, working memory, and cognitive flexibility), the effect of prenatal SRI exposure was markedly different in 6-year-old children depending on the child's *SLC6A4* genotype and mother's concurrent mood. SRI exposed children with an LL genotype showed pronounced differences in their EFs depending on their mother's current mood. SRI-exposed children with at least one short *SLC6A4* allele showed resilience (no impairment in inhibition and attention). Even in the face of more symptomatic mothers, the accuracy of the ≥1 S children on the difficult mixed Block 3 of the H&F test did not differ. In contrast, children with two L alleles, were far more sensitive to the context of life with a depressed mother. When their mother had few or no depression symptoms, LL children did extremely well—no other group regardless of *SLC6A4* genotype or mother's mood had mean scores that were as high. However, when their mothers were highly symptomatic, they performed worse than any other group including ≥1 S children with equally symptomatic mothers and LL children with less symptomatic mothers.

Differences in accuracy were most evident on the most-demanding EF Block. In general EF differences between groups often emerge only when cognitive skills are pushed to their limit. Children's EFs were not significantly affected by the child's mood (anxiety or depressive symptoms), though in this cohort there was little variation in the children's subclinical mood symptoms. Parents reported fewer inattentive and externalizing behaviors in children with prenatal SRI exposure regardless of the child's genotype. That might be because the SRI improved the child's postnatal environment (by improving the mother's mood) or because the effect on the 5HT signaling in the child secondary to prenatal SRI exposure. The benefit of one S allele of *SLC6A4* to EFs (cognitive control, self-regulation, inhibitory control) in children prenatally exposed to an SRI antidepressant became most apparent when children were in a particular environment (i.e., when their mothers were relatively more depressed). In that environment, the effects of the LL exposed children suffered but the effects of the exposed with ≥1 S allele did not. In this way maternal depression might act as a “prism,” dramatically increasing variability in EFs, according to prenatal SRI exposure and allelic variation.

Critical to identifying the impact of prenatal SRI exposure, is distinguishing the effects of the antidepressant from the maternal mood disturbance (pre and postnatal) that resulted in antidepressant medication use. Sensitivity to maternal depressed mood and its impact on cognitive development has been widely reported across childhood (Gelfand and Teti, 1990; Goodman and Gotlib, 1999; Elgar et al., 2004; Gross et al., 2008). Long before birth, early life influences are already shaping core cognitive capacities that go on to become critical for learning and mental health during childhood (Kolb et al., 2003; Fox et al., 2010). Preterm birth (Davis et al., 2011), prenatal psychological distress (Buss et al., 2011) and maternal behavioral risks (smoking, alcohol use, drug use) (Espy et al., 1999; Schonfeld et al., 2006; Blood-Siegfried and Rende, 2010) exert an influence on early EFs. Early and chronic exposure to maternal symptoms adversely affects early development of EFs (Hughes et al., 2013). Yet, not all outcomes on cognitive developmental pathways are necessarily negative in this setting (DiPietro et al., 2006), raising critical questions of how maternal mood affects cognitive development and who remains at risk, even in the presence of maternal pharmacotherapy. In the present study, prenatal SRI exposed children with at least one S allele showed stable EF functioning regardless of whether their mother was more or less depressed. Moreover, maternal mood in the present study, when their children were 6 years old, was mainly at a subthreshold level, well-below a typical DSM-IV criteria for Major Depressive Diorder (MDD; American Psychiatric Association, 2000), thus highlighting the importance of a spectrum of maternal mood symptoms on child development, rather than a clinical cutoff score.

Converging evidence also points to links between changes in 5-HT signaling and cognition in both animal models and humans (Munafò et al., 2009; Homberg et al., 2010), though not all studies have been consistent (Schmitt et al., 2006; Homberg and Lesch, 2011). Increased 5-HT signaling, secondary to SRI treatment and genetic variations, has been associated with improved cognitive functions. 5-HT transporter knockout rodent models, analogous to an extremely low activity (short allele) variant, have been associated with improved cognitive flexibility during reversal learning tasks (Brigman et al., 2010; Nonkes et al., 2012), as well as morphological frontal cortex changes reflecting an increase in central 5-HT levels (Jedema et al., 2009; Kalueff et al., 2010; Nonkes et al., 2010). Consistent with these findings, early developmental exposure to fluoxetine has been associated with improved spatial learning in rats (Bairy et al., 2006). In humans, carriers of the S allele showed improved performance on an attentional inhibition task (Roiser et al., 2007). Adults homozygous for SS alleles outperform LL carriers on cognitive tasks requiring inhibitory control, including episodic memory and attention (Roiser, 2011), reaction time (Enge et al., 2011) and executive attention (Strobel et al., 2007). Among S carriers, better performance on the Wisconsin Card Sorting test has been reported (Borg et al., 2009), reflecting the impact of increased 5-HT signaling on cognitive flexibility. In contrast, lower 5-HT levels also appear to impair reversal learning (Clarke et al., 2007). Cognitive consequences of increased 5-HT signaling associated with SRI antidepressant exposure have shown mixed results as well. In animal models, not all findings reflect the same impact on cognitive flexibility (Homberg et al., 2007b).

The neuroanatomical, and functional consequences of changing 5-HT levels depend on the timing (critical periods) and direction (increased or decreased) of the developmental exposure to changes in 5-HT signaling and may differ from the impact of an acute exposure in a mature organism. (Ansorge et al., 2007; Kalueff et al., 2010). In a rodent model, SRI exposure during a very specific postnatal period (postnatal days 4–21) of development is also associated, paradoxically, with reduced exploratory behavior, and depressive and anxiety-related behaviors in adulthood. These effects mimic the very effects of genetic 5-HTT inactivation (i.e., gene knockout models leading to the absence of the transporter); suggesting that increased serotonergic signaling during a developmentally critical period predisposes to subsequent affective disturbances (Lira et al., 2003; Ansorge et al., 2004, 2008). This central serotonergic auto-feedback hypothesis suggests that increased feedback signaling in the presence of high serotonergic tone blunts maturation of the 5-HT system via long-term developmental activation of inhibitory receptors (i.e., 5-HT_1a_), paradoxically leading to psychopathology later in life (Hensler, 2006; Ansorge et al., 2007; Simpson et al., 2011). While one might consider that maternal SRI treatment during pregnancy could potentially confer benefit on fetal neurodevelopment—via improved maternal mood—such exposure could also have detrimental effects later in childhood, reflecting a long-term consequence of decreased serotonergic tone. SRIs may elevate fetal 5-HT levels, but then ultimately lead to decreased 5-HT signaling later in life and restricted serotonergic system development. The serotonergic auto-feedback hypothesis, however, is not a unitary construct and further work is needed to understand how developmental changes in 5-HT signaling influences the downstream interaction with the social environment inherent to life with a depressed mother that together contributes to childhood behavior in this setting (Oberlander et al., 2009).

Importantly, not all factors that affect 5-HT signaling confer the same risk. Early life experiential variables influence susceptibility to environmental factors (Moffitt et al., 2005; Caspi and Moffitt, 2006) and not all outcomes associated with the short allele are necessarily negative (Risch et al., 2009). While adults with two short alleles may be at increased risk for depression (Caspi et al., 2003) following early life adversity, those raised in a nurturing environment may ultimately have a lower risk for depressive symptoms (Taylor et al., 2006). Increased central 5-HT associated with the *SLC6A4* short allele may therefore contribute to an increased sensitivity to environmental stimuli or hyper vigilance, leading to adaptation in one setting or an increased risk for poor mental health in another. In other words, in a low reward or low adversity setting, such hyper vigilance may confer an actual benefit that increases processing of relevant stimuli improving learning and social cognition (Homberg and Lesch, 2011). In the current study, serotonergic tone, via either prenatal SRI exposure or *SLC6A4* allelic variations, appeared to affect a self-regulatory capacity that might heighten sensitivity to a world with a depressed mother. Highly vigilant individuals may therefore either become vulnerable or resilient, depending on the demands of that social environment.

Our findings may also illustrate the influence of how allelic variations in the context of both early (i.e., fetal) and ongoing (i.e., postnatal/childhood) life experience shape a “biological sensitivity to context” (Boyce et al., 1995; Ellis et al., 2011) influencing adaptation and the diversity of child developmental outcomes following early changes in 5-HT signaling. This model proposes that phenotypic plasticity might enable a child to match their biological and behavioral capacities to the demands of their developmental environment. In this context, genetic variations may confer advantages for some children in supportive environments, but disadvantages for others who face social adversity in the context of maternal depression (Boyce and Ellis, 2005). Our findings showing higher accuracy in the non-exposed, LL children in the context of a minimally depressed mother, supports this claim.

Our findings point to a broader understanding of the impact of serotonin developmental neurobiology. While the “S” allele has been widely considered the “risk” or “sensitive” allele whereby the effect varies with context (Barr et al., 2004; Belsky and Pluess, 2009; Homberg and Lesch, 2011; van Ijzendoorn et al., 2012) our findings suggest that under certain circumstances carriers of the L allele may also be equally or even more sensitive to context. How this reflects the underlying changes in serotonin signaling (i.e., increased or decreased serotonin at developmentally sensitive times) remains a matter of speculation (Oberlander et al., 2009). Under some circumstances the L allele may confer vulnerability such as fear in adults exposed to carbon dioxide (Schruers et al., 2011) or aggression in 3-year old children of prenatally anxious mothers (Oberlander et al., 2010) when compared with LS or S allele carriers. Our findings take this observation one step further. Even with similar prenatal exposures, two children with different genetic inheritance show divergent developmental outcomes depending on the environmental circumstances they find themselves in at 6 years of age. Namely, while the impact of allelic variations may be environmentally dependent and the influence can, depending on the childhood context they grow into, go in both directions, thereby reflecting both developmental risk in some settings *and* resiliency in others. In this way, gene by environment outcomes may reflect a “conditional adaptation” (Boyce and Ellis, 2005) whereby allelic variations can be susceptible to both stressful and supportive contexts—for better and for worse (Belsky et al., 2007).

Conceivably there could be both advantages and disadvantages to improved EF performance. On one hand heightened vigilance may reflect an increased sensitivity in the social world of early childhood. However, it may also reflect a relative deficit in self-regulatory capacity which might illustrate a “leading edge” or susceptibility for a mood disorder that may emerge later in childhood (Taghavi et al., 1999). Interestingly, in a rodent model, an early increase in 5-HT signaling was associated with early fluoxetine exposure and paradoxically leads to increased anxiety and depression behaviors in adulthood (Ansorge et al., 2004). Earlier we reported that increased anxiety and depressive symptoms were observed by parents in 3 year olds with prenatal SRI exposure, though current increased maternal depression symptoms also contributed to child behavior (Oberlander et al., 2010). Now by 6 years of age, in the same cohort, levels of anxious behaviors did not differ between non-exposed children and fewer externalizing and attentional behaviors were observed in the exposed children. The long term implications of this unfolding longitudinal pattern remains unknown, however, improved EFs may reflect an endophenotype that includes increased vigilance that may evolve into a clinically apparent mood disorder in later childhood. Although increased vigilance may confer benefits for short-term tasks in one context (e.g., during a laboratory EF testing), it may be disadvantageous in the long run under other typical childhood circumstances (e.g., during an entire school day). While improved cognitive control in one setting may confer a developmental advantage (such as life with a depressed mother), the long term consequences of our findings in other childhood contexts (e.g., stressful classroom) need further study.

### Limitations

A number of limitations need mentioning. First, without direct measures of central changes in 5-HT signaling in utero and again at 6 years, we can only infer that prenatal SRI exposure and genetic variations did indeed alter 5-HT function accounting for our findings. Further, serotonergic system function is dependent on multiple neurochemicals, receptors and related genes, and a focus on prenatal SRI exposure and genetic variations for 5-HT transporters offer only a limited insight into a complex developmental system underlying early human cognitive development. Additionally, study of parent-child relationships which have been noted as key influences on individual differences in a developing child's executive capacities (Carlson, 2003; Hughes and Ensor, 2009; Bernier et al., 2011) are needed.

## Summary

This study sought to examine the long-term effects of prenatal SRI exposure on EFs at 6 years of age and to determine whether effects are moderated by maternal mood and/or genetic variations. For prenatally SRI-exposed children, regardless of maternal mood, accuracy of children with reduced 5HTT expression (at least one short [S] allele) remained stable regardless of maternal depressive symptoms. In particular, even with somewhat depressed mothers (though all symptoms were below clinical threshold), these children's EFs were comparable to children with the same genotype whose mothers showed few if any depressive symptoms—in this sense, they showed resilience. In contrast, children with two long (L) alleles appeared sensitive to context. When their mothers reported relatively fewer depressed symptoms, LL children showed extremely good EF performance—better than any other group. When mothers reported more depressive symptoms, LL children's EF performance was worse than that of any other group. Further, parents reported fewer inattentive behaviors in their SRI exposed children.

In the face of a mother with a relatively more depressed mood (albeit not at clinical levels), EFs were best preserved in children prenatally exposed to SRIs and with at least one short *SLC6A4* allele. Yet, prenatally-exposed LL children hold out promise of possibly superior EF if their mother's mood remains euthymic or improves. Together these findings may reflect effects of both increased and decreased early serotonergic signaling associated with an increased sensitivity to social and relational contexts. While improved EFs might reflect an apparent resiliency to an “at risk” social environment, the long term and clinical implications of these findings remain to be determined.

### Conflict of interest statement

The authors declare that the research was conducted in the absence of any commercial or financial relationships that could be construed as a potential conflict of interest.
